# Supplementary figures and images for: A spontaneous complex structural variant in rcan-1 increases exploratory behavior and laboratory fitness of Caenorhabditis elegans
Source: PLoS Genet. 2020 Feb 24;16(2):e1008606. doi: 10.1371/journal.pgen.1008606 (PMC7058356; doi:10.1371/journal.pgen.1008606)

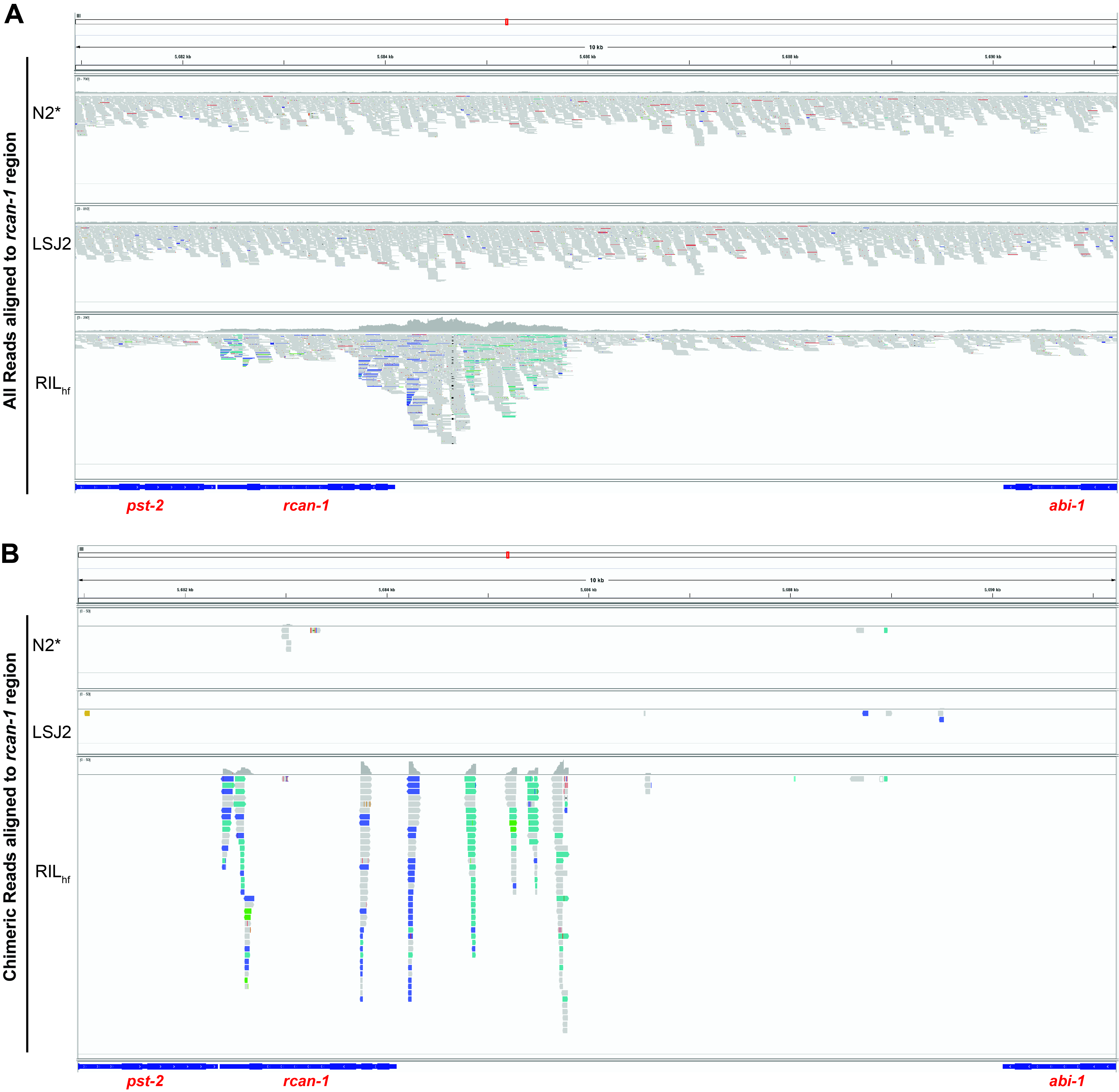

Supplement: S1 Fig — (A) IGV plot of illumina sequencing short reads align to rcan-1 genomic locations. (B) Chimeric reads align to rcan-1 genomic locations. Reads are from the resequencing of the N2*(CX12311), LSJ2, and RILhf (CX12348) strain. Besides an increase in coverage at the rcan-1 locus, a large number of chimeric reads (i.e. reads that partially map to two locations) were found in the RILhf strain. (Reads with grey color indicates they are normal reads (Pair orientations: LR); Reads with cyan color imply inversion (Pair orientations: LL); Reads with blue color imply inversion (Pair orientations: RR); Reads with green color imply duplication or translocation (Pair orientations: RL). Reads with red color have larger than expected inferred sizes.) (TIF) [file pgen.1008606.s001.tif]

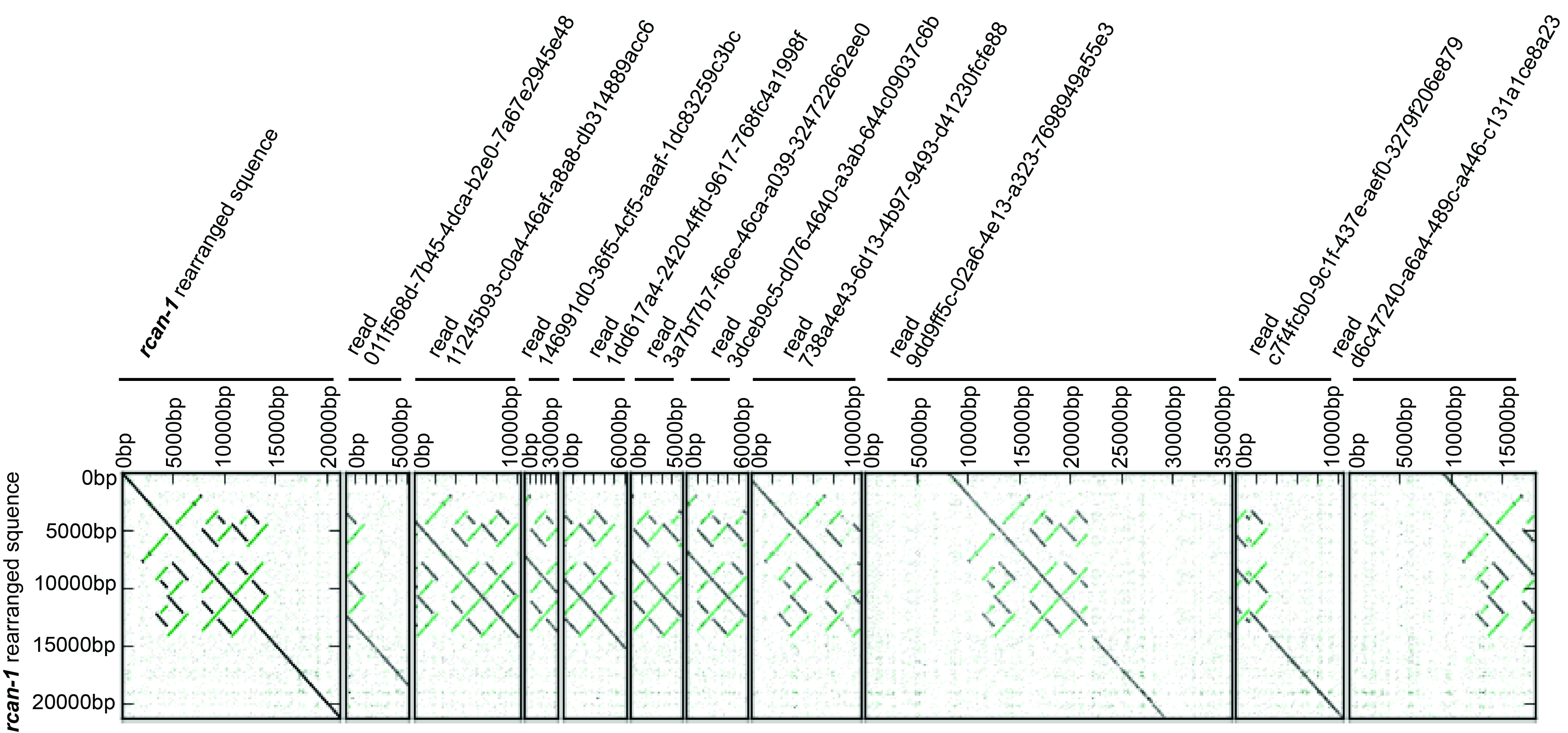

Supplement: S2 Fig — 10 nanopore sequencing reads that overlapped the rcan-1 structural variant were used to generate a dot plot with proposed rcan-1 rearrangement. (TIF) [file pgen.1008606.s002.tif]

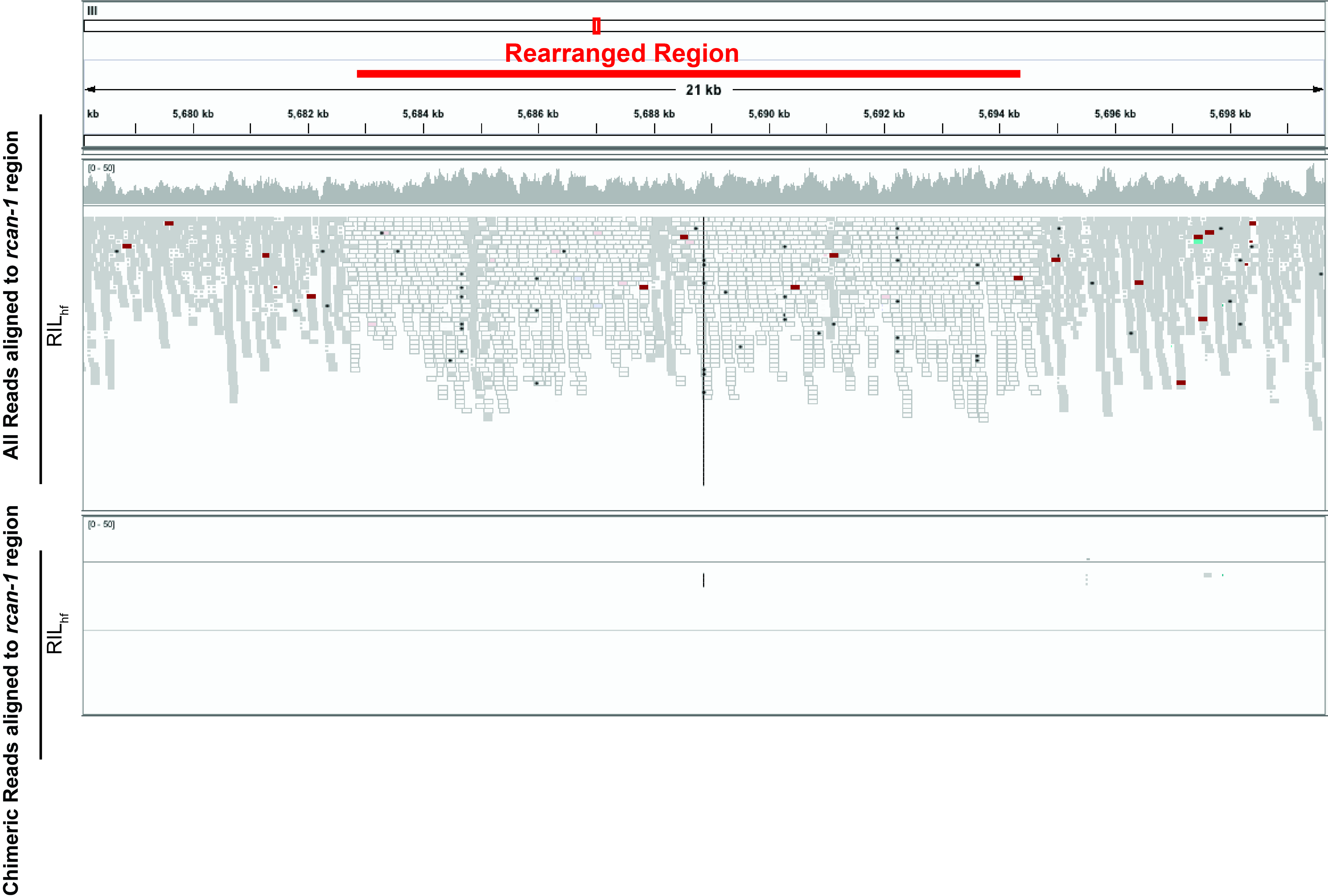

Supplement: S3 Fig — Top: All reads aligned to the rcan-1 rearrangement. Bottom: Chimeric reads aligned to the rcan-1 rearrangement. The uniform coverage and lack of chimeric reads is consistent with the proposed structure of the rearrangement. (Reads with grey color indicates they are normal reads (Pair orientations: LR); Reads with cyan color imply inversion (Pair orientations: LL); Reads with red color have larger than expected inferred sizes. Reads with empty color have low mapping quality.) (TIF) [file pgen.1008606.s003.tif]

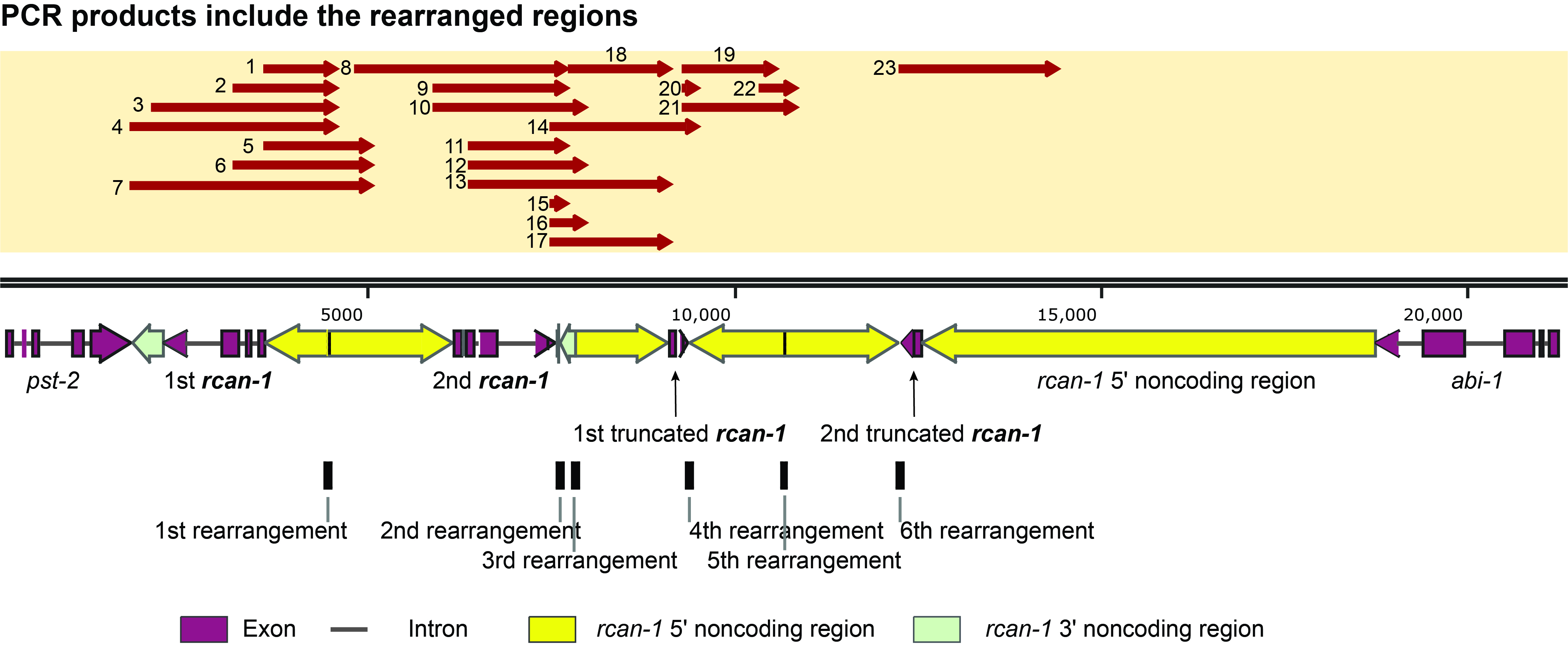

Supplement: S4 Fig — Red arrows are the PCR products that include the rearranged regions. The detail information of the primers, the expected length and observed length in agarose gel of each PCR product is listed in S2 Table. (TIF) [file pgen.1008606.s004.tif]

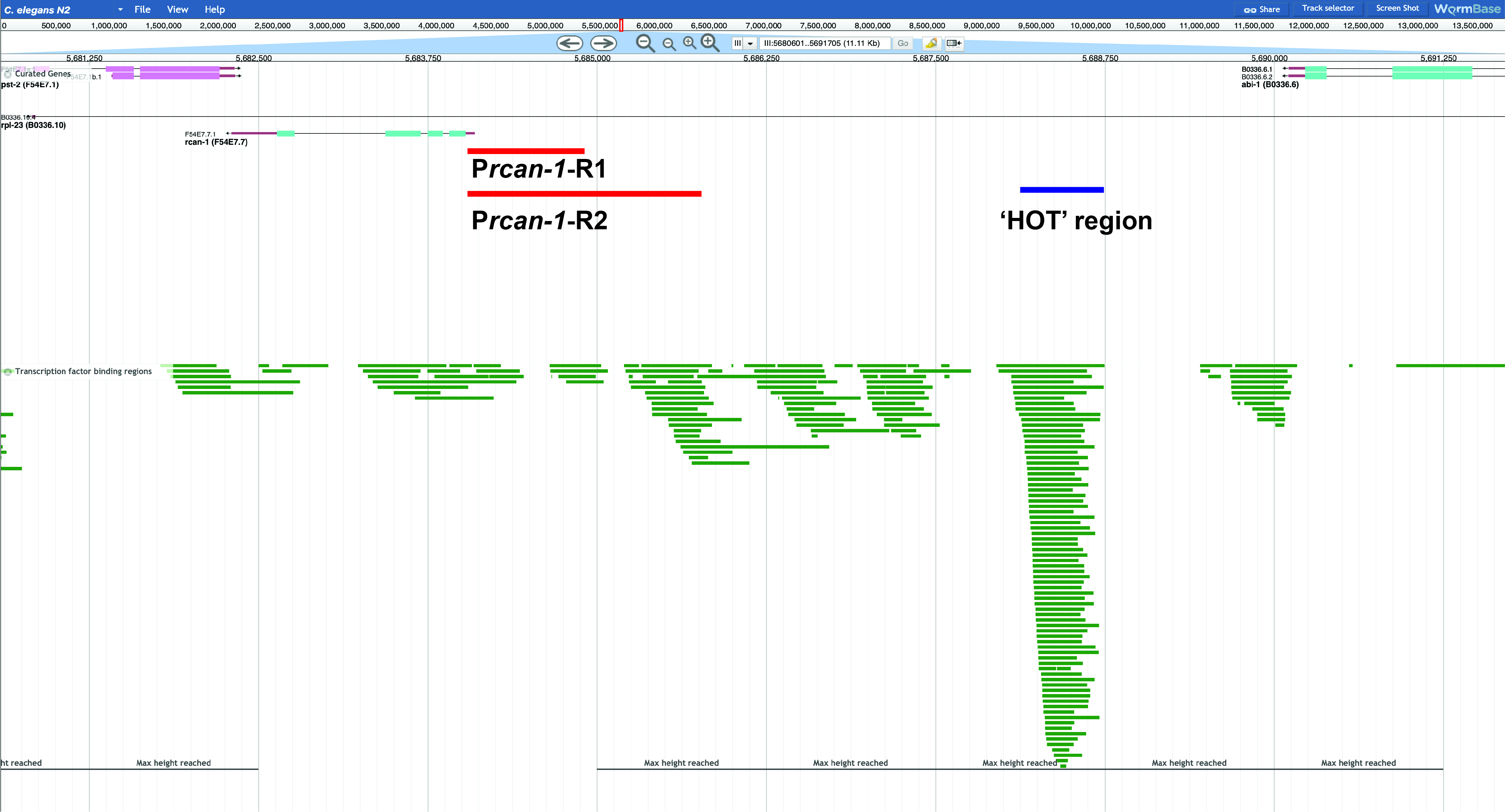

Supplement: S5 Fig — The green bars represent the transcription factor binding region. The red bars represent the two truncated promoter regions that drive full length of rcan-1 gene body in the complex rearrangement. The blue bar represents the highly occupied target region (‘HOT’). The figure is generated from Wormbase J-browser by adding the feature of transcription factor binding regions. The information of the transcription factors is listed in S4 Table. (TIF) [file pgen.1008606.s005.tif]

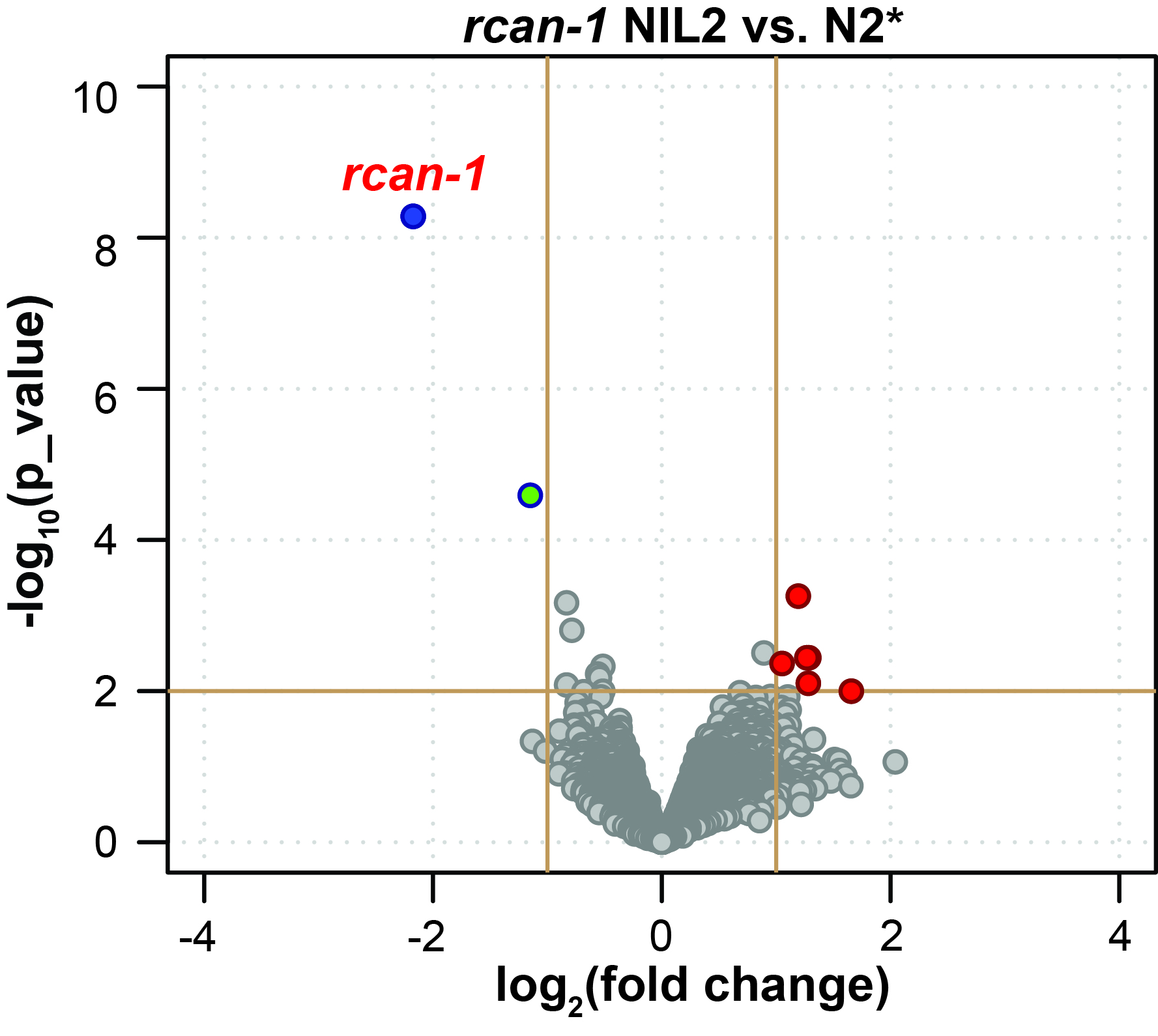

Supplement: S6 Fig — Red dots indicate genes with increased expression in rcan-1 NIL2 vs. N2* (p<0.01, log2(Fold Change) > 1). Cyan dots indicate genes with decreased expression in rcan-1 NIL2 vs. N2* (p<0.01, log2(Fold Change) < -1). The list of differential expressed genes with significance are available in S5 Table. (TIF) [file pgen.1008606.s006.tif]

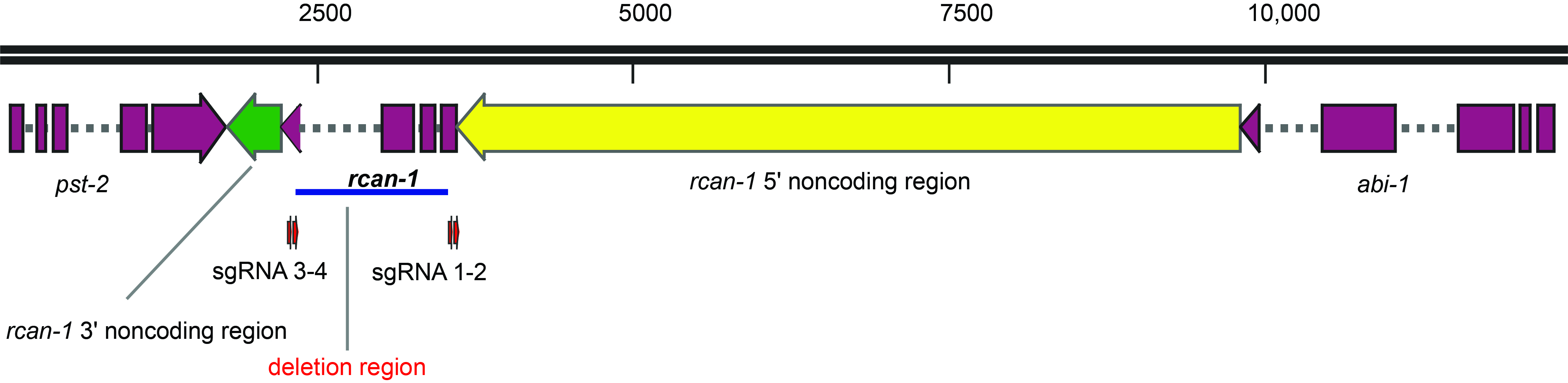

Supplement: S7 Fig — The position of two pairs of sgRNAs that target the 5’ and 3’ end of the rcan-1 coding region. The resulting deletion allele is shown as a blue box. (TIF) [file pgen.1008606.s007.tif]

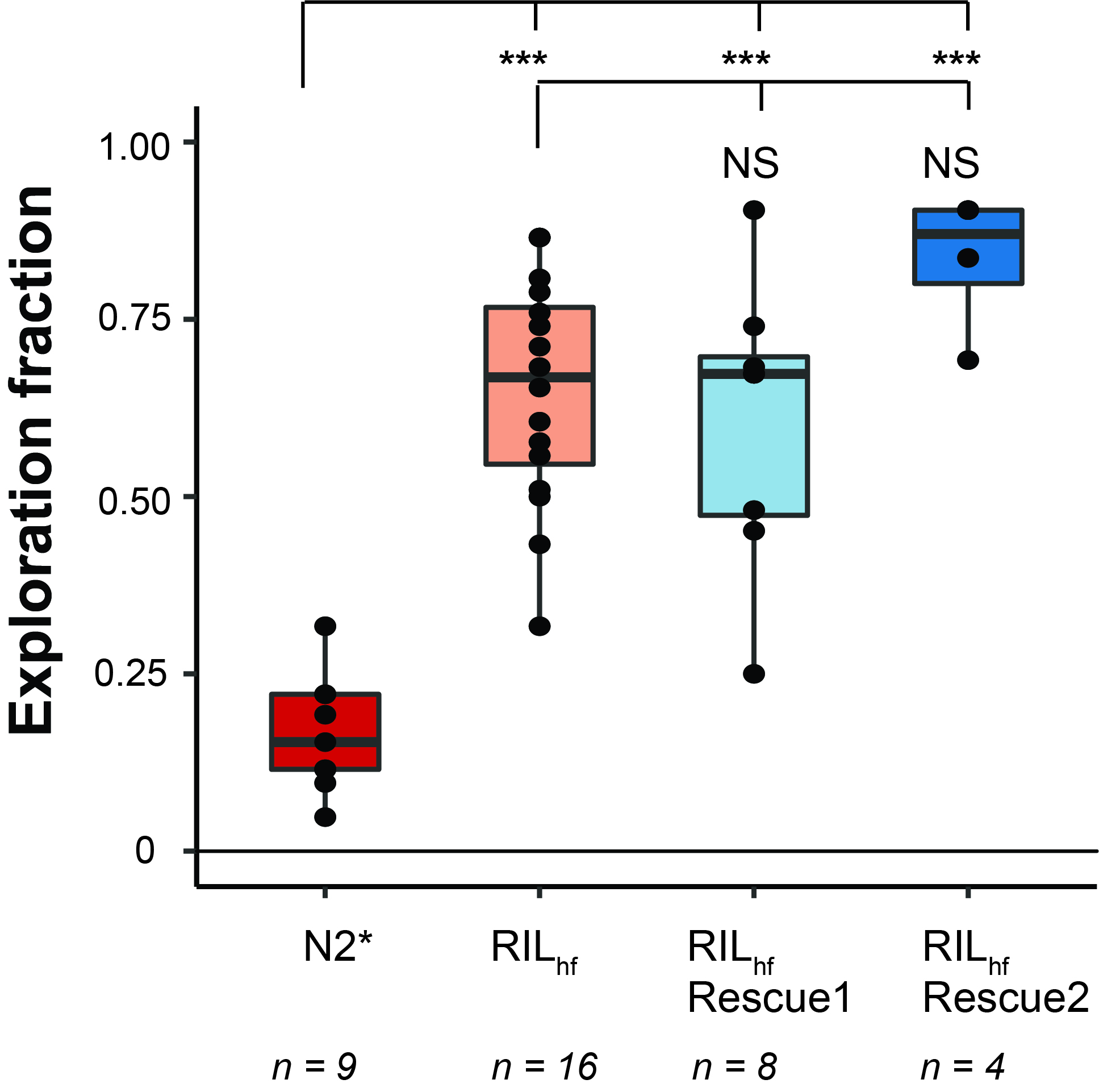

Supplement: S8 Fig — The RILhf animals were co-injected with 50ng/uL Prcan-1(4.5Kbps)::rcan-1 PCR product, 5ng/uL pCFJ90, and 45ng/uL pSM. The exploration fraction of the animals that express mCherry were measured. (TIF) [file pgen.1008606.s008.tif]

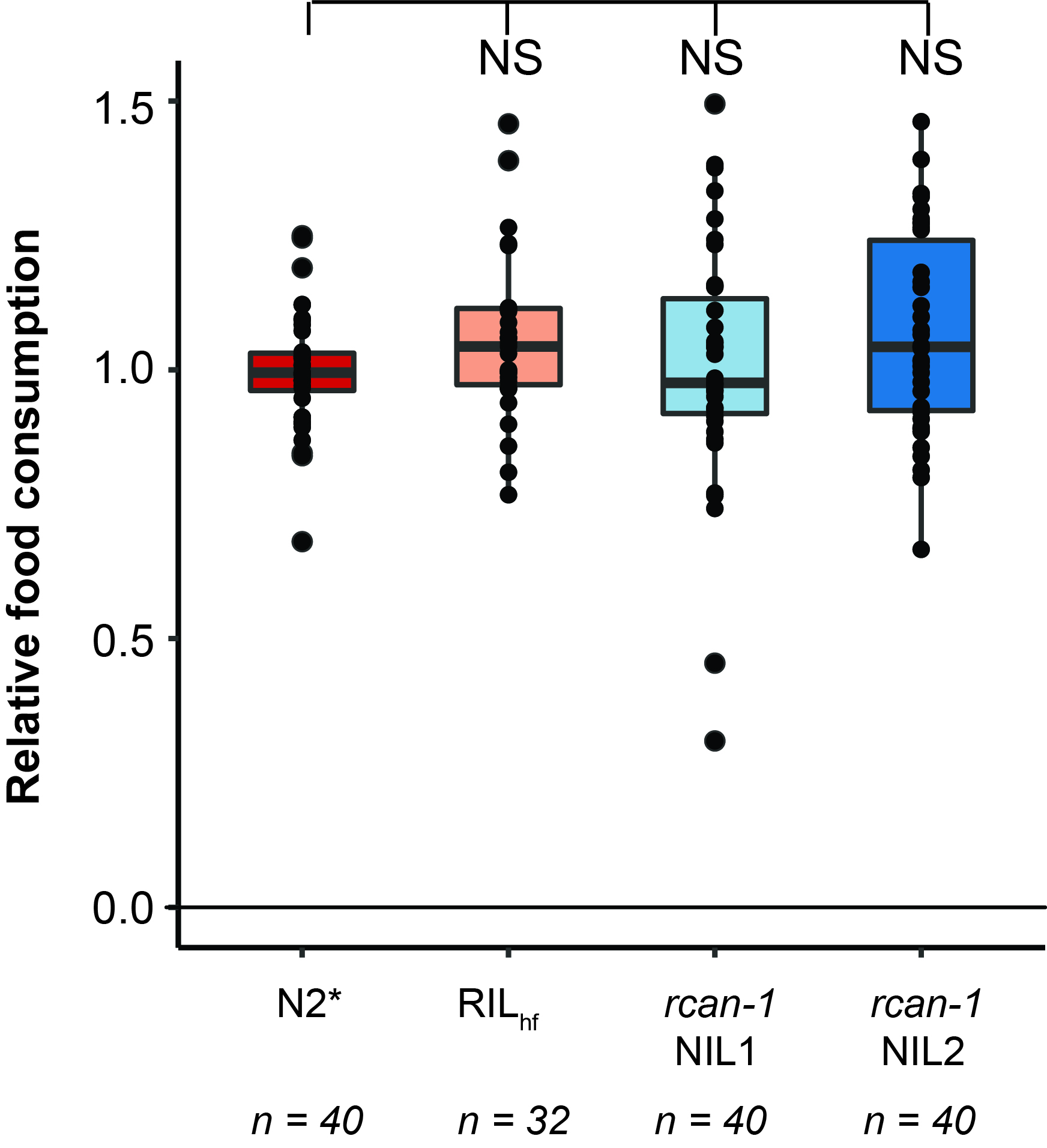

Supplement: S9 Fig — Relative food consumption of indicated strains. Each dot indicates one experimental replicate. (TIF) [file pgen.1008606.s009.tif]
