# Supplementary material for: A spontaneous complex structural variant in rcan-1 increases exploratory behavior and laboratory fitness of Caenorhabditis elegans
Source: PLoS Genet. 2020 Feb 24;16(2):e1008606. doi: 10.1371/journal.pgen.1008606 (PMC7058356; doi:10.1371/journal.pgen.1008606)
Supplement: Attachment — Submitted filename: ResponseToReviews.pdf [file pgen.1008606.s021.pdf]

First, we would like to thank the editor and the reviewers for their careful review of the paper. The reviews were uniformly helpful and fair.

In order to respond to the reviews in a timely matter (i.e. Minor revisions should be returned in one month), we have not performed every experiment requested by the reviewers. While we agree with the logic behind the requested experiments, performing all of the experiments would have required ~8 months of time due to some limitations in the competition experiments. It is probably useful to list some of these limitations explicitly:

While powerful, the competition experiments are difficult to perform in that they 1. Require a stable genetic line, so they cannot be performed on heterozygote animals or transgenics animals carrying extrachromosomal arrays (e.g. each generation the genotype of the strains will change). Some of the requested experiments would require generating integrated transgenes or further developing the competition assay to measure fitness of heterozygote animals. 2. Performing the competition experiment requires a decent amount of time. For example, for strains that already exist, it takes ~15 days to grow the animals for multiple generations without starvation, ~20-30 days of competition (5-7 generations), and then ~1 week to perform DNA extraction, quantification, digestion, ddPCR, and finally analysis on a large number of samples (multiple 96 well plates). 3) Finally, these experiments are relatively expensive (each replicate costs ~$25, mostly due to costs of the ddPCR).

Therefore, we have focused on the experiments that were in common between the three reviewers, namely:

1. Determining the fitness of the *rcan-1* deletion compared to the reference strain (Fig 7C).
2. Determining the fitness of the RIL_{hf} strain on uniform bacterial lawn plates (Fig 7D).
3. Determining the foraging phenotype of RIL_{hf}/NIL_{hf}/*rcan-1*$_{del}$ heterozygotes (Fig 7E)
4. Attempting to rescue the rearrangement with an overexpressed transgene.

With the exception of number 4, these experiments went largely as expected. We discuss these more fully in the point-by-point response to the reviews. We have added three additional supplemental figures and an additional main figure (figure 7 with some of the data from figure 6).

We also take the reviewers points that while the rearrangement of *rcan-1* alters its transcriptions, it is still speculative to say that the reduction in expression is responsible for the fitness gains. We make this clearer throughout the text. A tracked changes version of the manuscript is included to see all of the changes in text we have made.

Finally, since the first author (Yuehui Zhao) has graduated and started a postdoc, these experiments were performed by Lijiang Long, a graduate student in the McGrath lab. He has been added to the paper as an author for this work.

Reviewer 1:
In the process of identifying genetic variants that contribute to differences in fitness between laboratory-evolved *C. elegans* strains N2 and LSJ2, the authors observed that one recombinant inbred line (RIL) displayed higher fitness than either of the parental strains. This strain (RIL_{hf}) also displayed increased roaming behavior, which may contribute to its increased fitness. To identify the sequence variation in RIL_{hf} that is responsible for its increased roaming and fitness, the authors generated new RILS and mapped the variation to a region on Chromosome III. Through analysis of Illumina coverage depth followed by long-read Nanopore sequencing, the

authors identify a complex rearrangement in the *rcan-1* locus in RIL_{hf}. Through backcrossing the authors support their claim that the *rcan-1* rearrangement is the causative mutation. Finally, the authors show that this rearrangement results in reduced expression of *rcan-1*, and displays more pronounced phenotypes than a *rcan-1* loss-of-function allele.

This research will be of interest to readers of PLoS Genetics for multiple reasons. First, spontaneous genetic variants that increase *C. elegans* fitness are rare and inform our understanding of laboratory-based evolution. Second, RCAN-1 is a conserved regulator of calcineurin, and this unique allele will be of interest to neurobiologists that study the genetic pathways underlying foraging behavior. Finally, structural variants such as this complex rearrangement are often overlooked in mutational analyses, and this study provides an example of how to identify such mutations.

However the central claim of the paper – that reduced expression of *rcan-1* underlies the phenotypes of RIL_{hf} – should be strengthened prior to publication.

Major comments:

1. The central claim of the paper is that the complex rearrangement in *rcan-1* causes the increased fitness and exploratory behavior of RIL_{hf}. The authors' main evidence to support this claim is that backcrossed strains (NILs) that carry only the *rcan-1* rearrangement in an N2* background phenocopy RIL_{hf} (**lines 240-255**). However the data supporting the genotypes of these NILs (**Table S2**: Identified small genetic variants in RIL_{hf}, NIL1, and NIL2) raise a few questions:

    a. Based on the title of this table, we should see all variants in RIL_{hf} relative to the reference strain WS245. This will include variants that were contributed by LSJ2, as well as new variants that arose in generation of the RILs (akin to the *rcan-1* rearrangement). The expectation is that these variants will be absent from NIL1 and NIL2. However only one variant is listed in the RIL_{hf} column, and it is actually still present in the NILs. Can the authors please expand the table to include all loci that are variant in RIL_{hf}

    We added new supplemental tables and the previous TableS2 is TableS3 in this revised manuscript. The table is expanded and include all loci that are variant in RIL_{hf}.

    b. Why are the CB4856-derived mutations (e.g. *npr-1* and *glb-5*) not present in this table? They should be present in all strains listed, and appear when compared to reference genome WS245. If the authors have filtered the list in some way to remove these and other variants, that should be explicitly stated.

    The CB4856 SNVs at kyIR1(V, CB4856>N2), qgIR1(X, CB4856>N2) in N2* are filtered out from this table. The comment is added in table S3.

    c. 30/31 of the variants listed in this table are present in NIL1 and/or NIL2, but not in N2*, LSJ2, or RIL_{hf}. Is the interpretation that all of these 30 variants arose spontaneously in the process of backcrossing RIL_{hf}? The authors may want to comment on this.

We agree with the reviewer's interpretation. These 30 *de novo* mutations are most likely spontaneous mutations that occurred prior to the process of NIL construction. The comment is added in table S3.

2. To prove that *rcan-1* is the causal gene, the authors should try to rescue the RIL_{hf} strain with a transgene that over-expresses *rcan-1.* The authors show convincingly that the *rcan-1* rearrangement reduces *rcan-1* expression (**Figures 6A-C)**. Introducing a wild-type copy of *rcan-1* under its native promoter (or perhaps a heterologous promoter) should rescue the fitness and roaming phenotypes and would improve the manuscript.

   We agree that transgenic rescue of the RIL_{hf} phenotype would improve the manuscript. We amplified the wild-type region of *rcan-1* and injected it into the RIL_{hf} strain to create a transgenic strain. However, this extrachromosomal array failed to rescue the RIL_{hf} phenotype (**Figure S8**). There are a number of caveats that can account for this – we might not have included all of the transcriptional control regions in the amplified product (due to time constraints we did not determine if it rescued the *rcan-1* deletion), overexpression could have an opposite effect on roaming, or potentially the rearrangement causes additional expression that the wild-type transgene does not provide. While inclusion of this experiment would have improved the paper, we were unable to complete the experiment in a reasonable timeframe.

3. The authors generate a loss-of-function *rcan-1* allele using CRISPR, and while it does not phenocopy the *rcan-1* NIL perfectly, it is encouraging that with regards to the exploratory phenotype it's moving in the right direction (**Figure 6E)**. However when testing this null allele for a fitness difference, the authors only compete it against the *rcan-1* NILs (**Figure 6F**). The authors should also test the *rcan-1* null allele for a fitness difference <u>relative to N2*</u> to see if it provides some benefit in the competition assay. This comparison is absent from **Figure 6F** and would support the authors conclusions.

   We added the competition experiment using *rcan-1 ko* against N2*. The result is shown in Figure 7C. The *rcan-1* null allele was not significantly different from N2*. The trend is towards lower fitness than N2*, in agreement with our model.

4. The authors are careful not to state that the fitness advantage of RIL_{hf} is due to the increased roaming behavior, but this hypothesis is testable and would add to the impact of the paper. Two environmental tricks can be employed to reduce the effects of roaming: (1) the competition experiment could be performed with the *E. coli* lawn spread to the edges of the plate, or (2) the experiment could be performed in a hypoxia chamber which suppresses the bordering behavior of strains carrying wild *npr-1* alleles. (The authors do test for a difference in food consumption in **Figure S6** but this is in liquid, and does not completely reflect food consumption in a plate-based assay.)

   We performed the first requested competition experiment and added the result in Figure 7D. The fitness of the RIL_{hf} animals in the uniform lawn plates was still higher than the N2* animals, indicating that the bordering behavior cannot account for the entire fitness increase.

   Also, to clarify some misconception on the original food consumption assays, these were performed on an agar plate. We added more details to the methods to make this clearer.

Minor comments:

1. When characterizing the *rcan-1* NIL strains, the authors show that NIL1, NIL2, and RIL_{hf} display decreased body length (**Figure 5D)**. This is the first (and only) mention in the entire paper of RIL_{hf} having a body length phenotype and should be further explained. Is this phenotype also an emergent property of the RIL_{hf}? Data for the LSJ2 parental strain is not shown. Does the *rcan-1* CRISPR null also display a length phenotype? There have been some reports of *rcan-1* modulating body length which may be helpful (Li…Lee, Journal of Molecular Biology 2015 and Li…Lee, Molecules and Cells 2016). As it stands, this piece of data is a non-sequitur and does not add much to the argument of the paper.

   We believe that this data is relevant, as fitness-proximal phenotypes are often taken as a measure of fitness. It is reassuring that length is affected in these strains. However, the mapping between fitness proximal phenotypes (size, reproduction, etc) and fitness is still poorly understood. We have added these references to the text.

2. Citations #56 and #58 are identical (Xu…McGrath, bioRxiv 2019)

   Corrected

Reviewer #2: In this manuscript, Zhao et al. characterize a structural rearrangement in the rcan-1 gene in C. elegans, and link this genetic change to alterations in foraging behavior and fitness. First, they use a panel of N2/LSJ2 RILs to investigate how genetic changes impact fitness, using a competitive fitness assay. They identify a single strain that appears to be an outlier and then show that this strain also displays in increase in exploratory foraging behavior. They then use genetic mapping and sequencing to show that this strain has a de novo genetic change in the rcan-1 gene that is linked to the fitness and foraging phenotypes. Zhao et al. characterize the genetic change in rcan-1 and show that it is a surprisingly complex genomic rearrangement that arose during generation of the RILs. Genetic studies show that the change in rcan-1 is causal of the fitness and foraging phenotypes. Moreover, they find that the rcan-1 genomic rearrangement alters expression levels of rcan-1.

Overall, this is an interesting and convincing paper that should be of broad interest to the readers of Plos Genetics. This detailed study of a new genomic rearrangement and its strong ties to fitness and behavioral changes provides new insights into how structural variants cause phenotypic variability. I suggest just a few ways in which the manuscript can be improved and indicate the degree to which this would be preferred (vs. necessary), in my view.

1. The authors characterize the structural rearrangement in rcan-1 through long read sequencing and confirm that the Illumina sequencing aligns well to the proposed rearrangement. They indicate that they were unable to PCR the entire region. If possible, I'd still suggest a series of smaller PCRs aimed at confirming their proposed rearrangement. I realize this is non-trivial given the repetitive nature of the rearrangement, but perhaps primers could be designed to span the new sequences at breakpoints (i.e. right around the chimeric reads). Overall, they already have strong evidence for their proposed rearrangement, but this structural variant is the central point of the paper and it would be preferable to have overwhelming confidence in their interpretation.

We added the Figure S4 and Table S2 that contain PCR products that support the complex structure.

2. The authors provide a solid first pass analysis of how the rearrangement of rcan-1 causes the foraging and fitness phenotypes, by comparing the NILs to a null mutant and examining how the non-coding changes impact expression (using fluorescent reporters). However, this is obviously still not fully resolved. For example, do the two truncated rcan-1 copies have any functional roles? Is this simply a gene dosage effect (seems unlikely…)? There are further analyses that could provide insights: A) what is the phenotype of the heterozygous NIL? B) Do CRISPR induced frameshift mutations in each of the four ORFs that comprise the genomic rearrangement impact the NIL phenotype? If possible, I'd suggest further exploration of this topic, but I do not view it is absolutely necessary for publication (especially given the challenges of targeting these repetitive copies via CRISPR, etc).

We have added experiment A to the paper (Figure 7E). The heterozygous NIL had an intermediate phenotype to the homozygous NIL and N2* animals. Prior to publication, we attempted to perform the experiments requested in B. Unfortunately, we were unable to obtain these strains so we have not performed these requested experiments.

Minor points:

1. Is the distribution of datapoints in Fig. 3B bimodal, as would be expected from a single causal variant? It is difficult to assess this as presented. Please provide a histogram or something to that effect.

We have added the histograms in Fig.3B.

2. The images in Fig. 6C are rather small and difficult to assess. I'd suggest larger, high-quality images that give the reader a more complete view of sites of expression. In addition, providing a well-labeled image for each of the promoter regions used would be helpful for future mechanistic studies of rcan-1 function.

We have enlarged the image in Fig 6C for a more complete view.

Reviewer #3: In this manuscript, the authors investigate the genetic basis of increased fitness in a single C. elegans outlier recombinant inbred line (RIL) that has higher fitness than its parental strains. This higher fitness is correlated with increased exploration of a bacterial lawn. The authors provide compelling data that show that the major causative allele contributing to these phenotypes is a de novo complex structural rearrangement of the rcan-1 gene involving a series of tandem inversions/duplications that ultimately leads to reduced expression of rcan-1 due primarily to truncations of the rcan-1 5' noncoding region. Though the paper does not explore the specific cellular mechanisms by which this rearrangement and rcan-1 affect fitness/exploration, it is a very interesting and clearly-written study based on solid data and is appropriate for PLOS Genetics. In particular, the genetic and long-read sequencing data to map the rearrangement and determine its structure are very strong. We suggest a few experiments that could strengthen the paper, but do not feel that these are critical for publication.

Considerations:
1. One of the interesting conclusions of the paper is that even though the rearrangement creates two full-length copies of the rcan-1 coding sequence, there is actually reduced rcan-1

expression (~25% normal total levels). This is supported by solid RNA-seq data and expression reporters (Fig. 6). Given that the 5' regulatory regions of both intact rcan-1 ORFs are truncated, reduced expression makes sense. The simplest model is that the truncations remove important enhancers and these enhancers no longer function as well when present more distantly in the remaining intact 5' regulatory region. However, the authors push the data further to argue that there are tissue-specific effects on expression and that the two orientations of the inverted and truncated 5'region have different effects on expression. These latter two conclusions are not as well supported by the existing data that are based on expression reporters of the truncated/inverted 5' regulatory region in the two orientations (Fig. 6B-D). The problem is that these expression reporters are extrachromosomal arrays that are overexpressed and are often variable in structure and expression levels from line to line or even different animals of the same line and thus should not be used for the kinds of fine quantitative inferences the authors make, such as the head expression being more affected than the body, especially as these inferences are based on a very small number of lines. To be able to make such fine comparisons the authors should generate single-copy transgenes that are not prone to variability. Additionally, the images in Fig 6C&D do not clearly show the distinct and tissue-specific effects the authors claim, which could again be the result of analysis of a small number of selected lines. Higher magnification images with better quantitation of expression in specific cells of interest (such as the unaffected head neurons) could also help.

We suggest that the authors either repeat these expression experiments with single-copy transgenes or tone-down their conclusions (lines 337-349, especially lines 347-349). For the data in Fig. 6B, they should specify whether the ~30 animals analyzed come from one or more transgenic lines. Three lines of each are shown in the strain list, but it isn't clear whether the data come from all three lines. In the raw data (Table S1), please list the strain and allele # of each animal analyzed as well.

For reasons discussed above, we have not created any single-copy transgenic animals. As requested, we have toned down the conclusions throughout the paper. The main point we would like to make is that the rearrangement of the promoter region of *rcan-1* has led to global decreases in its expression. Whether these decreases are responsible for the fitness changes remains to be determined.

We have also made changes to Table S1 as requested

2. The authors provide good evidence that the residual expression of rcan-1 in the rearrangement is important for increased fitness by showing that near-isogenic lines (NILs) carrying this rearrangement outcompete a rcan-1 null mutant (Fig. 6F). The rcan-1 null has increased exploration (Fig. 6E) compared to a WT control, but it is unclear whether the null mutant also has increased fitness. To test this, a good experiment would be to compete the rcan-1 null vs. the WT control. This experiment would further address the argument that reduced expression of rcan-1 increases fitness and whether fitness is indeed tightly correlated with exploration.

We have added this data to Figure 7C.

It is clear that some of the complexity of the rearrangement is necessary for its fitness advantage, but it is unclear how much of the complexity is necessary. The simplest model is that the two intact rcan-1 ORFs with their altered upstream regulatory regions would be sufficient – that reduced rcan-1 expression plus some residual expression (possibly tissue-specific) is sufficient. To test this, a strain could be generated with the two altered 5' regions R1

and R2 driving the rcan-1 ORF (as single-copy transgenes to control expression levels) in the background of the rcan-1 null mutant. Would this strain fully recapitulate the phenotypes of the more complex rearrangement lines?

We agree that these experiments would be informative but we did not perform these due to time constraints.

Other simple experiments could further probe the relationship between rcan-1 dosage and exploration/fitness and strengthen the conclusions. The rearrangement has about 25% normal total expression of rcan-1, though this may be higher in some cells and lower in others. What is the phenotype of a rcan-1(lf)/+ heterozygote that would be predicted to have 50% rcan-1 levels in all cells? What is the phenotype of the rearrangement when heterozygous? Is the rearrangement rescued (i.e. fitness and exploration decreased) by injection of WT rcan-1(+)?

We have tested the heterozygotes (Figure 7E). These experiments suggest that there is a strong relationship between rcan-1 dosage and exploration behavior, as the heterozygotes were intermediate to the parental strains.

We also attempted to rescue the exploration behavior using a RILhf strain carrying an extrachrosomal transgene constructed from a PCR product amplifying the wildtype *rcan-1* region. This transgene was unable to rescue the exploration behavior. There are a number of reasons that could account for this negative result: 1) we did not amplify enough of the upstream or downstream region to capture full expression, 2) The rearrangement leads to expression changes that are not captured in the wildtype promoter, or 3) Overexpression of *rcan-1* reduces foraging behavior. We include this data in the paper (Figure S8), however, since it is difficult to interpret a negative result, we do not discuss it extensively.

3. The NILs are shown to be sufficient for increased exploration and fitness (Fig. 5). But do they fully account for the increased fitness of the original RILhf strain? To test this, a good experiment would be to compete the NILs with RILhf.

While this experiment would be interesting for suggesting follow up experiments, we did not perform this experiment due to time/expense discussed in the beginning of this document,.

4. On several occasions, it is suggested that variation on chromosome V contributes to the exploration phenotype of RILhf (e.g. lines 190, 261). Though the LSJ2/RILhf RILS clearly show an effect of chromosome V (LSJ2 alleles on this chromosome are associated with increased exploration), it seems that variation on this chromosome can contribute to exploratory behavior, but it seems unlikely that such variation is contributing to the phenotype of RILhf since it has no LSJ2 alleles on chromosome V (perhaps RILhf would explore even more with LSJ2 alleles on chromosome V, but the assay used here is already maxed out so wouldn't be able to resolve this). More likely, alleles on LSJ2 chromosome V have increased exploration independently of the rest of the RILhf genetic background, a possibility that could be easily tested if desired. However, this is really a fairly minor point and tangential to the paper. We suggest just making it clear in the writing that chromosome V variation may affect exploration, but not necessarily the exploration phenotype of RILhf.

We agree, and have made changes to the discussion.

5. Some simple investigations into the modified regulatory sequence would be interesting. Are there known transcription factor binding sequences or conserved sequences in the 5' noncoding

region that are lost in the truncations?

We have added Figure S5 and TableS4 detailing our analysis of previously published ChIP-seq experiments showing that the upstream region of rcan-1 contains a large number of transcription factor binding sites.

6. A deeper discussion of worm exploration behavior could enrich this manuscript. What is known about the circuitry controlling this behavior? Is rcan-1 expressed in specific relevant neurons (and expression lost in the R1 and R2 constructs)? Additionally, it is ultimately unclear how exploration relates to fitness and whether this correlation is causal or coincidental. Throughout the paper, we are led to believe that the altered exploration could lead to increased fitness by changing how worms feed (the exploration behavior is sometimes called a “foraging strategy” but perhaps more care should be taken with these terms). But late in the discussion (line 405), we learn that there is no difference in food consumption in strains carrying the rcan-1 rearrangement. An interpretation of this surprising result seems warranted. Does this mean that exploration is unrelated to food consumption (and fitness) or might there be other ways that exploration is related to fitness? The authors don’t need to have the answers or take a side, but it would be nice to have some conclusion since it was just confusing to provide this result with no further comment. (Perhaps not calling exploration a foraging strategy would be advisable, especially when increased exploration is considered “increased foraging activity” as in line 363).

We have performed an experiment on uniform bacterial plates (Figure 7D), which showed the RILhf strain was still more fit than the N2* strain. In general, it is very difficult to determine the exact phenotypes responsible for fitness changes. It is indeed surprising that food consumption is not different between the two strains. We have added some additional speculations to the discussion as requested.

7. Is the known functional connection of rcan-1 to tax-6/calcineurin important? If rcan-1 inhibition of calcineurin is relevant to its exploration and fitness phenotypes, then it might be expected that there is increased calcineurin activity in strains with the rearrangement, and a tax-6 mutation would suppress the increased exploration and fitness phenotypes of the rearrangement.

Our working model is that rcan-1 acts through tax-6, however, we have not tested this yet.

8. There’s a fair bit of discussion of the rcan-1 rearrangement being formed as an adaptation in response to selective pressures (lines 35-38, 368-385, 393-395). Though the rearrangement clearly increases fitness, it does not seem that it was selected for increased fitness, but rather was likely just formed and fixed accidentally. In fact, the way the RILs were made, there did not seem to be any obvious selection for fitness since about half of the lines have low fitness like their parent LSJ2 (Fig 1D). We recommend a more conservative discussion of these points.

We have made these changes.

Minor points:
1. The authors are to be commended for using box plots rather than bar graphs in the figures (and for providing the raw data in a supplemental file), but the figure legends should give n values for quick reference.

We added the number of replicates under the box plot for reader’s quick reference.

2. It is unclear why the authors measured animal length (Figure 5D). This is the only time we

hear of this phenotype in the paper and it is unclear if it is considered to be an important contributor to increased fitness. As presented, the experiment doesn't really seem to fit in the paper, but is just a random piece of data thrown in.

We believe that demonstration that this rearrangement affects a fitness-proximal trait is important to the manuscript. The length was measured with a COPAs high-throughput sorter, and analysis of this data includes regressing away certain confounding variables. We have modified the text to make this clearer.

3. For Fig. 6A and S4, it would be nice to know which other genes have significantly altered expression (the green and red dots), and whether the same genes were affected in both NILs. This information is not easily derived from the raw data in Table S3.

The previous TableS3 is TableS5 in this revised manuscript. We have added this information to TableS5 sheet2.

4. The way the Fig 6A legend is written, it is unclear whether this figure shows combined data from the two NILs ("differences between the NIL and N2* strains"). Given that the figure itself is labeled NIL1, we presume that Fig 6A shows the data from NIL1 and Fig S4 shows the data from NIL2. Please make this clear.

Corrected.

5. The Fig 6A legend should say what the green and red dots mean. The Fig 6C&D legend should say what the arrowheads indicate. It would be best if all the images in Figs 6C and 6D are shown with the same anterior-posterior orientation and mention this orientation in the legend. It seems that the zoomed-in images in 6D show different animals than in 6C, but this should be made explicit.

Corrected.

6. In Figure 4A, the schematic for the gene position is difficult to interpret. It would be good to color code rcan-1 differently from pst-2 so it can be seen where these genes start and end, and show the direction of transcription of each gene (as in Fig 4C). In fact, we recommend using the same schematic in 4C and 4A so that they can be directly compared, especially for the 5' noncoding region. A scale bar for Fig 4A would also help so we can more easily see how much of the 5' region has increased coverage.

Corrected.

7. In Figure 6B, the significance bars for statistical comparisons are difficult to interpret, especially the top two levels of these comparisons. It is unclear which data sets are indicated as significantly different from each other. For instance, what do the two *** values at the top refer to? The head of WT compared to head of R1 and R2, the body of WT compared to the body of R1 and R2, etc? Or head, body, and total of WT compared as a combined group to head, body, and total of R1 and R2?

Corrected

8. It would be interesting to see if there are differences in movement speed between the N2*,

RILhf , and rcan-1 NILs. This may be linked to exploratory behavior.

While we agree this would be interesting, given the uncertain importance of the behavior to the fitness gains, we have not performed this experiment.

9. The description of the structural changes in the rcan-1 rearrangement is useful but could be condensed. The paragraph at the bottom of page 9/top of page 10 repeats many of the same things said in the opening paragraph of page 9 where the rearrangement is first described.

Corrected

10. There should be explanation for what the different colors mean in Figures S1 and S3.

Corrected

11. The acronym “CNV” should be defined when first used in the abstract.

Corrected

12. In Table S3, rcan-1 is written as rcn-1. Please correct this to facilitate searching for the data.

Corrected

13. Line 93 should read: “in wild strains of C. elegans” instead of “in wild strains C. elegans.”

Corrected

14. There is inconsistency on whether the rearrangement carries five or six tandem inversions (e.g. lines 25 and 213).

Corrected

15. Line 366. The rearrangement is described as “similar” to a previously published rearrangement that causes another phenotype in C. elegans. However, it’s not clear what is meant by “similar” and this other rearrangement is in fact quite different. It consists of several duplications and a triplication, but no inversions, and causes a phenotype through increased gene dosage rather than decreased expression as reported here. Additionally, the other rearrangement seems to be formed by chromoanasynthesis in which the rearrangement is made by templated synthesis and the breakpoint junctions have short microhomologies. This seems quite different from the rearrangement described here, which does not seem to have any microhomologies at the breakpoints (though this could be explicitly mentioned). The only similarity seems to be that complex rearrangements can cause phenotypes, but we would argue that the rearrangements themselves are not similar at all.

We have corrected the text to make this clearer

16. Lines 383-385: the point of this sentence is unclear.

We have removed this sentence

17. The paragraph in lines 407-412 seems overly speculative.

We have toned this paragraph down

18. References 56 and 58 are the same (and can be updated now that the paper is published).

Corrected.

Reviewed (and signed) by Michael Ailion and Lews Caro
