# Supplementary material for: A spontaneous complex structural variant in rcan-1 increases exploratory behavior and laboratory fitness of Caenorhabditis elegans
Source: PLoS Genet. 2020 Feb 24;16(2):e1008606. doi: 10.1371/journal.pgen.1008606 (PMC7058356; doi:10.1371/journal.pgen.1008606)
Supplement: Attachment — Submitted filename: Review.docx [file pgen.1008606.s020.docx]

In the process of identifying genetic variants that contribute to differences in fitness between laboratory-evolved *C. elegans* strains N2 and LSJ2, the authors observed that one recombinant inbred line (RIL) displayed higher fitness than either of the parental strains. This strain (RIL_hf_) also displayed increased roaming behavior, which may contribute to its increased fitness. To identify the sequence variation in RIL_hf_ that is responsible for its increased roaming and fitness, the authors generated new RILS and mapped the variation to a region on Chromosome III. Through analysis of Illumina coverage depth followed by long-read Nanopore sequencing, the authors identify a complex rearrangement in the *rcan-1* locus in RIL_hf_. Through backcrossing the authors support their claim that the *rcan-1* rearrangement is the causative mutation. Finally, the authors show that this rearrangement results in reduced expression of *rcan-1*, and displays more pronounced phenotypes than a *rcan-1* loss-of-function allele.

This research will be of interest to readers of PLoS Genetics for multiple reasons. First, spontaneous genetic variants that increase *C. elegans* fitness are rare and inform our understanding of laboratory-based evolution. Second, RCAN-1 is a conserved regulator of calcineurin, and this unique allele will be of interest to neurobiologists that study the genetic pathways underlying foraging behavior. Finally, structural variants such as this complex rearrangement are often overlooked in mutational analyses, and this study provides an example of how to identify such mutations.

However the central claim of the paper – that reduced expression of *rcan-1* underlies the phenotypes of RIL_hf_ – should be strengthened prior to publication.

Major comments:

1. The central claim of the paper is that the complex rearrangement in *rcan-1* causes the increased fitness and exploratory behavior of RIL_hf_. The authors’ main evidence to support this claim is that backcrossed strains (NILs) that carry only the *rcan-1* rearrangement in an N2* background phenocopy RIL_hf_ (**lines 240-255**). However the data supporting the genotypes of these NILs (**Table S2**: Identified small genetic variants in RIL_hf_, NIL1, and NIL2) raise a few questions:
a. Based on the title of this table, we should see all variants in RIL_hf_ relative to the reference strain WS245. This will include variants that were contributed by LSJ2, as well as new variants that arose in generation of the RILs (akin to the *rcan-1* rearrangement). The expectation is that these variants will be absent from NIL1 and NIL2. However only one variant is listed in the RIL_hf_ column, and it is actually still present in the NILs. Can the authors please expand the table to include all loci that are variant in RIL_hf_?
b. Why are the CB4856-derived mutations (e.g. *npr-1* and *glb-5*) not present in this table? They should be present in all strains listed, and appear when compared to reference genome WS245. If the authors have filtered the list in some way to remove these and other variants, that should be explicitly stated.
c. 30/31 of the variants listed in this table are present in NIL1 and/or NIL2, but not in N2*, LSJ2, or RIL_hf_. Is the interpretation that all of these 30 variants arose spontaneously in the process of backcrossing RIL_hf_? The authors may want to comment on this.
2. To prove that *rcan-1* is the causal gene, the authors should try to rescue the RIL_hf_ strain with a transgene that over-expresses *rcan-1.* The authors show convincingly that the *rcan-1* rearrangement reduces *rcan-1* expression (**Figures 6A-C)**. Introducing a wild-type copy of *rcan-1* under its native promoter (or perhaps a heterologous promoter) should rescue the fitness and roaming phenotypes and would improve the manuscript.
3. The authors generate a loss-of-function *rcan-1* allele using CRISPR, and while it does not phenocopy the *rcan-1* NIL perfectly, it is encouraging that with regards to the exploratory phenotype it’s moving in the right direction (**Figure 6E)**. However when testing this null allele for a fitness difference, the authors only compete it against the *rcan-1* NILs (**Figure 6F**). The authors should also test the *rcan-1* null allele for a fitness difference relative to N2* to see if it provides some benefit in the competition assay. This comparison is absent from **Figure 6F** and would support the authors conclusions.
4. The authors are careful not to state that the fitness advantage of RIL_hf_ is due to the increased roaming behavior, but this hypothesis is testable and would add to the impact of the paper. Two environmental tricks can be employed to reduce the effects of roaming: (1) the competition experiment could be performed with the *E. coli* lawn spread to the edges of the plate, or (2) the experiment could be performed in a hypoxia chamber which suppresses the bordering behavior of strains carrying wild *npr-1* alleles. (The authors do test for a difference in food consumption in **Figure S6** but this is in liquid, and does not completely reflect food consumption in a plate-based assay.)

Minor comments:

1. When characterizing the *rcan-1* NIL strains, the authors show that NIL1, NIL2, and RIL_hf_ display decreased body length (**Figure 5D)**. This is the first (and only) mention in the entire paper of RIL_hf_ having a body length phenotype and should be further explained. Is this phenotype also an emergent property of the RIL_hf_? Data for the LSJ2 parental strain is not shown. Does the *rcan-1* CRISPR null also display a length phenotype? There have been some reports of *rcan-1* modulating body length which may be helpful (Li…Lee, Journal of Molecular Biology 2015 and Li…Lee, Molecules and Cells 2016). As it stands, this piece of data is a non-sequitur and does not add much to the argument of the paper.
2. Citations #56 and #58 are identical (Xu…McGrath, bioRxiv 2019)
