# Supplementary material for: A spontaneous complex structural variant in rcan-1 increases exploratory behavior and laboratory fitness of Caenorhabditis elegans
Source: PLoS Genet. 2020 Feb 24;16(2):e1008606. doi: 10.1371/journal.pgen.1008606 (PMC7058356; doi:10.1371/journal.pgen.1008606)
Supplement: Attachment — Submitted filename: ResponseToReviews.docx [file pgen.1008606.s022.docx]

We again thank the reviewers and editor for their review of our manuscript. We have attempted to take all their comments into account and change the manuscript in a way that would satisfy their concerns. A tracked changes file is included to see all changes to the text. Detailed responses to each point follow below.

**Reviewer #1**: The authors have identified an interesting laboratory-derived strain (RILhf) with a spontaneous mutation that causes increased fitness and roaming behavior. My primary concern with the manuscript was that the claim that the rcan-1 rearrangement is causal for the increased fitness and roaming behavior needed to be strengthened. The authors have attempted to address this concern, and the revised Table S3 is excellent and addresses my questions about the backcrossed NILs.

However it is disappointing that rescue of RILhf with wildtype rcan-1 did not work, with respect to foraging behavior (Figure S8). In the author’s “response to reviewers” this experiment is interpreted clearly, but I have some concerns with the authors interpretation in the paper (lines 300-302). My concerns are as follows:

“potentially this indicates that the rearranged region causes ectopic expression necessary for the changes in foraging behavior…” – this is inconsistent with the rcan-1 CRISPR KO allele promoting foraging behavior.

“…or overexpression of rcan-1 decreases foraging behavior” – this is the opposite of what the data is showing, so perhaps a typo? Do the authors mean overexpression of rcan-1 might increase foraging behavior? Otherwise you would have expected the construct to rescue, since RILhf displays increased foraging behavior.

We agree that the lack of rescue is disappointing and that the paper interpretation is confusing and have changed it to the following:

“Potentially, the transgene does not fully recapitulate wildtype expression of *rcan-1,* potentially lacking upstream elements required for expression in cells necessary for the changes in the exploration behavior. Alternatively, additional genetic variants in RIL_hf_ also promoter exploration behavior independent of the *rcan-1* rearrangement.”

I think more likely is this particular transgenic experiment didn’t work, either for technical reasons, or because additional variations in RILhf promote foraging behavior independent of the rcan-1 rearrangement. Nevertheless, the manuscript deserves to be published in PLoS Genetics, as the rcan-1 rearrangement is a very complex mutation, and fully dissecting the genetics of the various rcan-1 duplications is outside the scope of this paper. Additionally, the findings are interesting and novel, and the experiments are performed carefully and not over-interpreted.

Typo in line 94: “in certain context”

Corrected

Typo in lines 238-239: should delete “enhancers and other r promoter regions are probably present in the truncated promoters”
Corrected

**Reviewer #2:** The authors have addressed all of my concerns.

**Reviewer #3:** I support publication of this paper, which has been significantly strengthened in this revision. The authors have done a very good job of responding to and addressing the majority of the critiques. Though I do not have major concerns, a few of my original points have not been fully addressed and I repeat them here. Also, many of the parts of the manuscript that were changed upon revision are not written with the same quality as the rest of the manuscript. I point out a number of clear grammar mistakes and typos, but suggest the authors look again at the writing in all their new or edited sections and work more carefully on these parts.

Points:

1. The authors have done a better job of toning down their conclusions about tissue-specific effects of the altered rcan-1 promoter regions in some places and pointing out some of the caveats of extrachromosomal arrays, but there are other places where they still explicitly state that there are tissue-specific effects: Fig 6 title (line 456), line 116, lines 277-279. I am fine with the authors suggesting there might be tissue-specific effects, but think they should not flat out state it as if it were demonstrably proven because the evidence is quite weak.

We have removed conclusions about tissue-specific effects from the manuscript.

2. I strongly recommend that the authors not use the term “foraging behavior” for the exploration phenotype and not equate increased exploration with “increased foraging activity.” It isn’t exactly clear what the increased exploration of rcan-1 variants indicates and whether it is causally related to the increased fitness, but it does not seem clearly related to foraging for the rcan-1 variants as their increased exploration does not lead to increased food consumption. Thus, it would be more conservative to just call it “exploration” as that is what is being assayed. The word foraging means “searching for or obtaining food.” The evidence given is that rcan-1 does not affect “obtaining food” and I don’t think it is addressed whether the exploration of rcan-1 variants indicates “searching for food.” An alternative unexplored possibility is that the rcan-1 variants may simply have increased locomotory activity or decreased reversals. Though increased exploration may be a foraging strategy for other mutants, the evidence in this paper does not suggest it is for the rcan-1 variants. Thus, continued use of the term foraging to describe this behavior is both potentially confusing and misleading. Examples include: the short title of the paper, line 51, lines 168-172, line 254, lines 293-308, lines 315-316, lines 349-353, and several Figure legends, but there may be others.

We have changed foraging behavior to exploration behavior throughout the manuscript.

3. Related to point 2, I think the food consumption experiment would fit better in the Results, in the same section with the experiment using uniform bacterial lawns, as a general consideration of the question of how exploration relates to foraging.

We have added this to the Results section where the reviewer suggests.

4. Though the authors now state that the rcan-1 rearrangement was unlikely to have been selected for its higher fitness, the sentences in lines 38-41 and 346-348 still make it sound that this rearrangement formed as an adaptation in response to selective pressures. I would tone these down.

We have removed all suggestions that this complex rearrangement was selected in the RIL panel due to its effects on fitness. However, we have left in discussion that the complex change and abstract COULD be selected for by positive selection (indeed it was in competition experiments).

5. I previously suggested that the paragraph in lines 371-376 is overly speculative and the authors responded by saying that they have toned this paragraph down, yet it reappears exactly the same as in the original.

We apologize for this mistake. We have modified this paragraph a bit to tone down its speculation, however, we prefer to keep this paragraph in the discussion. While speculative, we think that it is an interesting direction to think about and we have attempted to rewrite this to make this clearer why.

6. I did not realize that body size is considered a fitness proximal trait, and am surprised that a smaller size would be associated with increased fitness (if anything, I would have thought the opposite). Thus, the experiment on body length could be better explained and motivated for naïve readers like me. For me, it still seems to come out of the blue, and the immediate segue from short body to increased fitness (line 230) is not intuitive at all.

We agree that the connection between fitness-proximal traits and fitness is not direct. It is not obvious to us either how fitness-proximal traits map onto fitness. This is likely environment specific. However, as phenotypes are acted on by selection, we think it is important to include this data in the paper. We have added a few lines to improve the transition to this data.

7. In Figure 5D (body length), the y-axis is unclear. It shows “normalized body length” but the units are unclear and it is unclear what it is being normalized to – the legend says “controls” but it is unclear what these controls are and there are no apparent data for any control strains in the Table S1 source data file.

We modify the Figure 5D legend and add more details to explain how each animal’s body length is normalized.

8. Figure 6C&D: please indicate anterior-posterior direction of these worms.

We add the markers in Figure 6C&D to indicate anterior-posterior direction of these worms.

9. The rcan-1 rearrangements suggests a simple and interesting model for the evolution of gene duplicates that wasn’t clearly presented in the discussion of this phenomenon (lines 321-338). How do gene duplicates evolve from redundancy to become paralogs? The rcan-1 rearrangement suggests a model that by imperfectly duplicating or rearranging the regulatory regions of the duplicated genes, two new genes can be created in a single step that aren’t functionally redundant because they may have differential expression levels and differential tissue-specific expression. Thus, this mechanism might create paralogs instantaneously without having to first evolve through redundancy.

We have modified the discussion to make this point clearer. We agree though that this is an interesting counter model that are data suggests.

Typos/grammar:

line 49: understanding

Corrected

line 167: causal

Corrected

line 230: these data (should be plural)

Corrected

line 237: should be “upstream sequence”

Corrected
lines 237-242: run-on sentence (also kind of unclear, I have a feeling it may have changed in ways unintended)

Corrected

line 238: r at end of line

Corrected

line 281: fluorescence

Corrected

line 307: solely

Corrected

lines 317-318: I think the cited paper has just one rearrangement, so shouldn’t be plural

The cited paper only contains one rearrangement. We corrected “rearrangements” to “rearrangement”.
line 319: unclear antecedent of “this.” Just say: “The rcan-1 rearrangement”

Corrected
line 335: grammar

Corrected
line 337: RILhf

Corrected
line 342: “single single-nucleotide” is awkward

Changed to “a single SNV”
line 378: described

Corrected
line 378: should be “often” instead of “common”

Corrected
line 466: compared

Corrected
lines 467-468, 470-471: grammar problems. Also, say what the white arrows in the R2 panels are showing.

We added the explanation of white arrow in Figure6C bottom panel that indicates the expression of mCherry driven by *P_rcan-1-R2_* in the head *region.* We removed the white arrow in Figure6D bottom panel.

lines 460 & 1045: Table S5

Corrected

lines 1053 & 1076: grammar problems

Corrected

Table S3 title: says Table S2 by mistake

Corrected

Figure S4: rearranged

Corrected

Figure S7: “deletion” is written on top of “region”
Corrected

Reviewed (and signed) by Michael Ailion.
